# The Autonomic Progress Bar Motivates Treatment Completion for Patients of Stimulant Use Disorder and Cannabis Use Disorder

**DOI:** 10.3389/fpsyt.2019.00944

**Published:** 2020-01-13

**Authors:** I-Chun Chen, Gloria Teng, Chur-Jen Chen, Tsuo-Hung Lan, Hung-Jen Liu

**Affiliations:** ^1^ Department of Psychiatry, Taichung Veterans General Hospital, Taichung, Taiwan; ^2^ Ph.D. Program in Translational Medicine, National Chung Hsing University, Taichung, Taiwan; ^3^ Department of Mathematics, Xiamen University Malaysia, Selangor, Malaysia; ^4^ Department of Applied Mathematics, Tunghai University, Taichung, Taiwan; ^5^ Faculty of Medicine, National Yang-Ming University, Taipei, Taiwan; ^6^ Institute of Molecular Biology, National Chung Hsing University, Taichung, Taiwan

**Keywords:** mandatory treatment, progress bar, motivation, stimulant use disorder, time series analysis

## Abstract

**Background:** The intrinsic motivation behind the “need to complete” is more influential than external incentives. We introduced a novel progress-bar tool to motivate the completion of programs designed to treat stimulant and cannabis use disorders. We further examined the effectiveness of the progress bar's scoring approach in forecasting consistently negative urine tests.

**Methods:** This study's participants included 568 patients with stimulant, amphetamine-type, and cannabis use disorders who were undergoing 12-month mandatory treatment programs at Taichung Veterans General Hospital in Taiwan. Patients were given scores of 1, -1, or 0 depending on whether they received negative, positive, or missing urinalysis reports, respectively. The autonomic progress bar generated weekly score totals. At the group level, score_i_ donated scores from all patients for a given week (i denoted the week). Score_i_ was standardized to adjusted score_i_. We then conducted Autoregressive Integrated Moving Average (ARIMA) Model of time-series analyses for the adjusted score_i_.

**Results:** A total of 312 patients maintained treatment progress over the 12-month program. The autonomic score calculator totaled the shared achievements of these patients. The coefficients of the lag variables for mean (p), lag variables for residual error term (q), and number of orders for ensuring stationary (d) were estimated at p = 3, d = 4, and q = 7 for the first half of the treatment program, and were estimated at p = 2, d = 2, and q = 3 for the second half. Both models were stationary and tested as fit for prediction (p < 0.05). Sharply raised adjusted scores were predicted during the high-demand treatment phase.

**Discussion:** This study's novel progress-bar tool effectively motivated treatment completion. It was also effective in forecasting continually negative urine tests. The tool's free open-source code makes it easy to implement among many substance-treatment services.

## Introduction

Artificial intelligence advancements have enabled unprecedented reforms in the domain of medicine. These advancements have recently expanded to the addiction treatment field. In this context, both computer and mobile-based applications have helped eradicate the care gap while removing treatment barriers. Such tools also provide cognitive behavioral training, automated newsletter reminders, and treatment motivation (1–6). Many mobile apps have successfully been used to augment alcohol abuse treatment (4), while computers have become major intervention and meeting tools among therapists who need to monitor patient progress in cases of depression and marijuana use disorders (7). Further, several studies have reported that patients with stimulant use disorders require the ability to monitor their treatment progress online; such an environment offers enhanced motivation (8–11).

Progress bars are used as percentage-completion indicators (8). They are widely used in several software contexts, including program downloading, online gaming, and data transmission. In this regard, studies have found that the “need to complete” provides motivation (9, 10). From the psychoanalytic perspective, addicts cast powerlessness to therapists, who then produce a sense of powerlessness to form projective identities. As such, progress itself is seen as a reward (11, 12). We believe that progress indicators create intrinsic motivation through the “need to complete” and that that such urgency is more influential than external incentives.

If compulsory treatment is synonymous with the unwillingness to receive treatment, then it is unsurprising that psychotherapy resistance is profoundly manifested by many patients. Although difficult at times, mandatory treatment is thus becoming more common, especially because it increases treatment adherence. Mandatory treatment requires patients to follow prescribed treatment plans that sometimes require attendance schedules and scheduled urinalyses. We believe that 12-month treatment programs can achieve greater success if used in conjunction with effective computer software designed to facilitate psychological operations through the “need to complete.” This will stimulate intrinsic motivation, thus prompting patients to adhere more closely to prescribed treatment plans. This study was comprised of two parts. First, we implemented a novel progress-bar tool to motivate treatment completion among patients of a 12-month program. Second, we examined the effectiveness of the progress bar's scoring approach (see *Measurements*) in forecasting continually negative urine tests.

## Methods

### Samples and Materials

**Figure 1:**
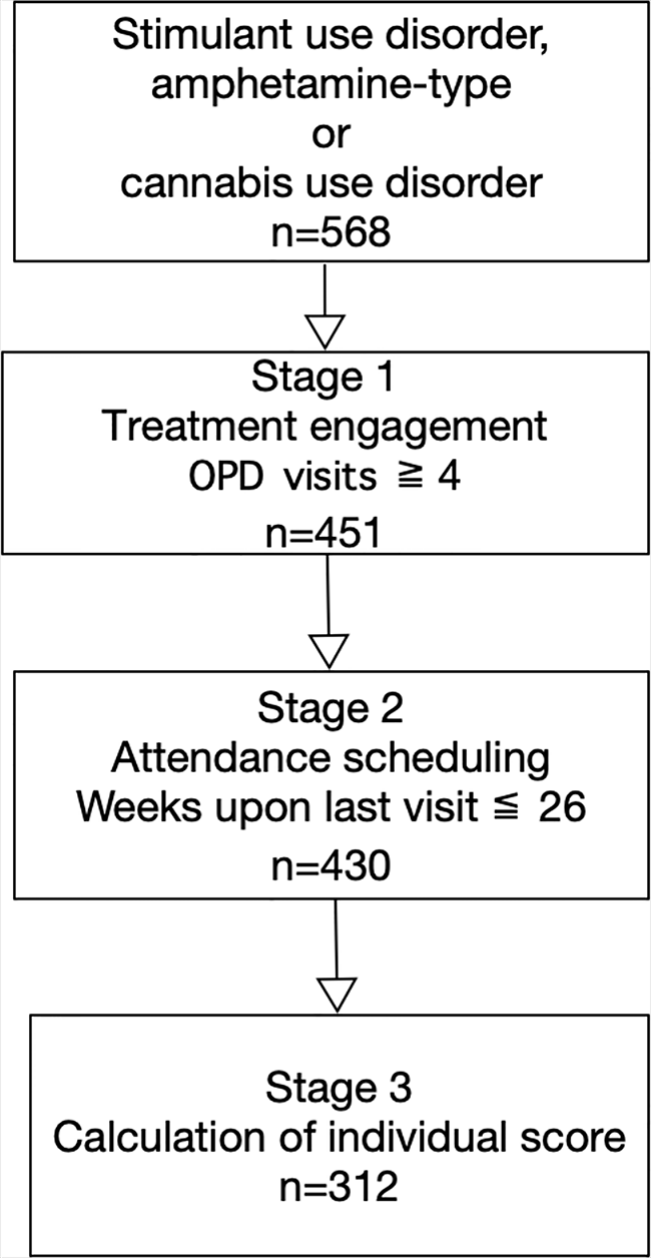
Study flowchart.

This was a retrospective follow-up study. Participants included 568 patients who were diagnosed with stimulant use, amphetamine-type, and/or cannabis use disorders between January 2013 and December 2018. All patients were required to complete 12-month treatment program at the Taichung Veterans General Hospital in Taiwan. All patients in this study were subjected to mandatory treatment. For inclusion in this study, patients were required to be at least 20 years of age and diagnosed with stimulant use, amphetamine-type, and/or cannabis use disorders based on diagnostic criteria of Diagnostic and Statistical Manual of Mental Disorders 5th Edition, DSM-5. We excluded patients who had completed fewer than four outpatient department visits upon treatment engagement. This ensured an adequate observation period for the time-series data. We also excluded patients whose most recent visit was more than 26 weeks before the study period. This was because confounding factors may have increased since that time. We also considered that missed treatments periods exceeding 26 weeks may have been due to factors related to the judicial system (e.g., patients had been rearrested and were required to start treatment anew). Those patients throughout the 12-month treatment program were selected to calculate the individual score (**Figure 1**). This study was approved by the ethics review committee at Taichung Veterans General Hospital (IRB number: CF18105A).

### Treatment Protocol

The substance treatment service at Taichung Veteran General Hospital was based on an adapted protocol-driven design. This ensured robust treatment boundaries from a psychological perspective. Patients were required to complete the treatment intervention according to an attendance schedule and were subjected to both urinalysis and group psychotherapy. Biweekly urinalyses were conducted to detect amphetamine, 3,4-Methylenedioxymethamphetamine (MDMA), and marijuana based on immunoassay during the first phase. For a given patient, stabilization was defined when a total of four negative urine samples had been observed. This was defined as the high-demand treatment phase.

Patients that completed the high-demand phase then transitioned to the low-demand phase. At that time, urinalysis was switched to a monthly basis. The average time at which patients began the low-demand phase was 6 months from treatment initiation. Here, each patient also attended a total of 10 biweekly group psychotherapy sessions. A positive urinalysis result required the respective patient to return to the biweekly urinalysis program.

### Measurements

We developed a scoring system to summarize and visualize patient progress. Each patient received a score of 1 for each respective negative urinalysis result. However, each positive urinalysis resulted in a score of -1. Finally, patients received scores of 0 for each missed urinalysis. The program generated weekly score totals and plotted respective curves for each patient.

**Figure 2:**
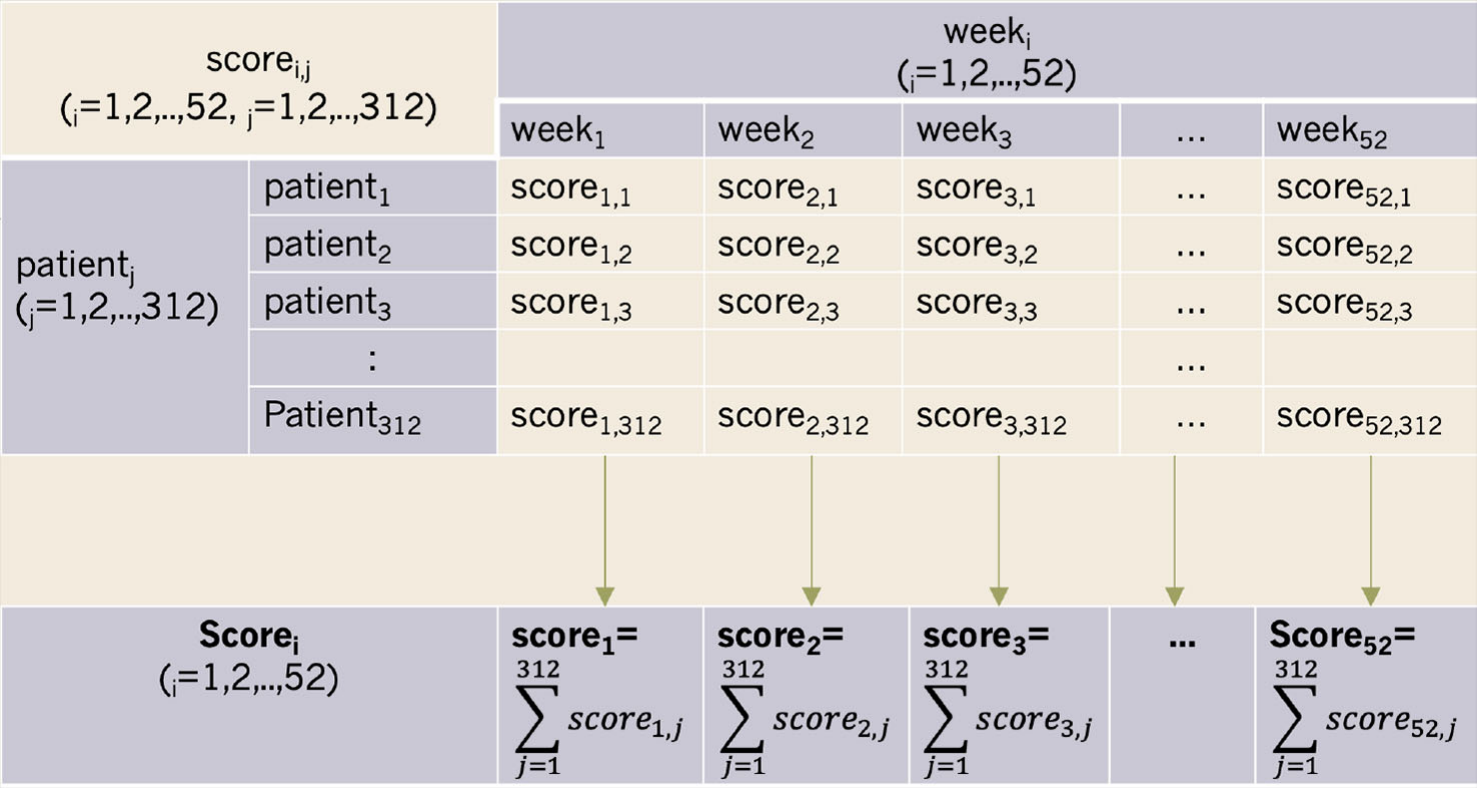
The definition of score_i_ in this autonomic progress bar.

This scoring program consisted of a score and algorithm. At the group level, score_i_ donated the total score from all patients in a given week (i denoted the week) (**Figure 2**). However, the number of patients who took urinalyses varied each week. As such, score_i_ was standardized to the adjusted score_i_. Adjusted score_i_ was then calculated as score_i_/n, where n denoted the number of patients who took urinalyses during week_i._ The open-source code for the progress bar is offered for free in the **Supplementary Material** section at the end of this document.

### Statistical Analysis

The adjusted score_i_ for each week during the 12-month treatment period was used to generate time-series data. The milestone for reaching the low-demand treatment phase (see *Treatment Protocol*) was usually achieved approximately six months after beginning treatment. As such, two trend components were adapted at the 27th week as a cut-off point. We determined whether the time-series data deviated from the white noise assumption by implementing an Autoregressive Integrated Moving Average (ARIMA) Model, while an augmented Dickey-Fuller single root test was conducted to test stationarity. We also estimated the coefficients of the lag variables for mean (p), lag variables for residual error term (q), and number of orders for ensuring stationary (d). Finally, we used the Box-Pierce test to determine whether the model was sufficiently robust for prediction. The R software (version 3.4.4) package was used for all statistical analyses. Differences were considered significant at p < 0.05.

## Results

Of the 568 total patients, 117 failed treatment engagement as defined by attending less than four outpatient visits. Of the remaining 451, 430 maintained their expected attendance schedules. As such, 312 patients appeared consistently throughout the 12-month treatment program (**Figure 1**). Among these, 97.5% had stimulant use disorder, of which 37.0% used amphetamine-type stimulants more than 4 days per week. Of the abovementioned 312 patients, 79.1% were male (mean age of 36.0± 8.7 years), while 17.3% also had alcohol use disorder, and 10.1% had opioid use disorder. Overall, 8.0% were men sexed with men, and 7.8% were HIV-positive. Further, 10.6% had psychotic disorders, and 1.6% with bipolar disorder (**Table 1**).

**Table 1:** Descriptive data of 312 patients of stimulant use disorder or cannabis use disorder completing 12-month treatment.

Baseline	Mean ± SD	N(%)
Male		247(79.1)
Age	36.0±8.7	
Stimulant use disorder		305(97.5)
Amphetamine use ≧ 4 times/ week		115(37.0)
Alcohol use disorder		54(17.3)
Opioid use disorder		31(10.1)
Men sexed with men		25(8.0)
HIV positive		24(7.7)
Psychotic disorder		33(10.6)
Bipolar disorder		5(1.6)

**Figure 3:**
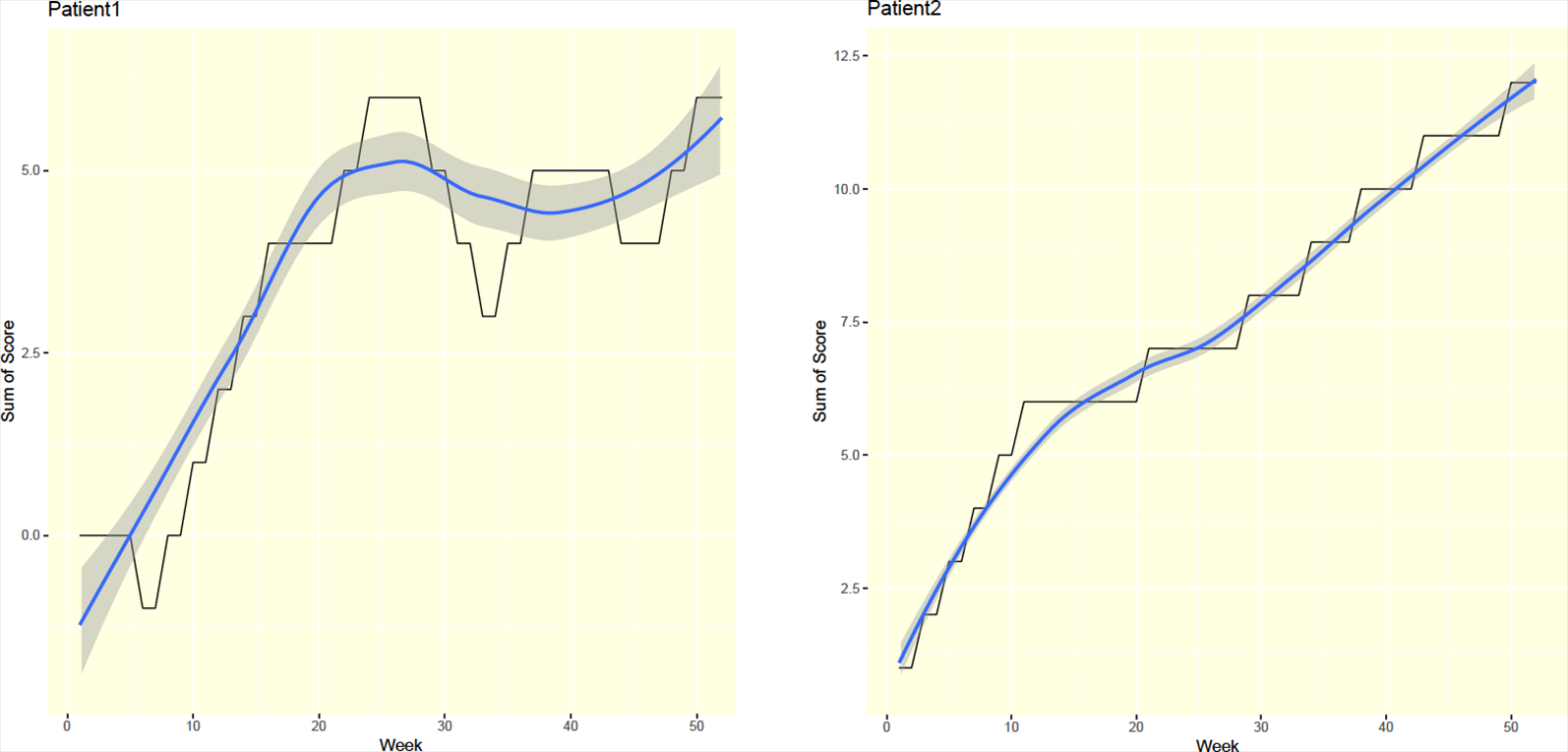
Two examples of patient's progress bar.

For each patient, this study's progress bar generated a total weekly score through a user-friendly graphical interface. That is, the application functioned as an autonomic progress indicator. For example, a steadily rising curve followed by a sharp fall may have reflected relapse, while a steadily increasing accumulated score likely reflected abstinence throughout the treatment course (**Figure 3**).

**Figure 4:**
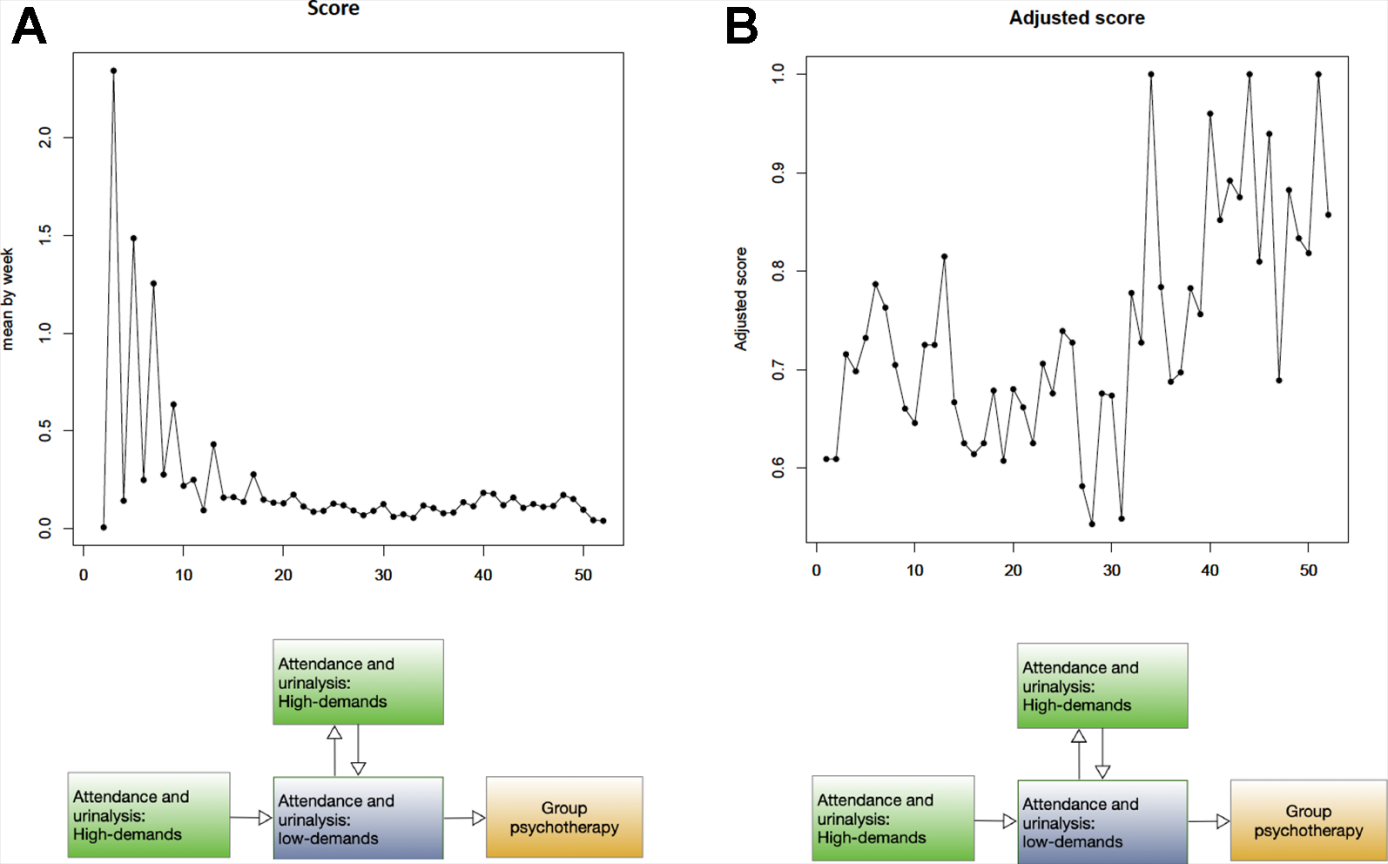
Time-series plot of overall 312 patients **(A) X**-axis: week, **Y**-axis: score **(B) X**-axis: week, **Y**-axis: adjusted score.

The progress bar facilitated patients in maintaining the vigilance needed to achieve their aims. In this context, we considered the addiction treatment service as an entity comprised of two halves. The first half was high-demand, while the second was low-demand. Patient progress during each half was measured according to score_i_, which denoted the total scores from all patients in a given week (i denoted the week). **Figure 4** shows overall weekly patient performance throughout the 12-month program. Here, the crude scores reveal larger fluctuations during the initial stages.

Total scores were dependent on the number of patients who completed urinalysis. Because the treatment protocol followed a biweekly schedule, the number of patients doing so was higher during weeks 2, 4, 6, and 8 than during weeks 1, 3, 5, and 7 (**Figure 4**). Given that the number of patients who completed urinalysis varied each week, score_i_ was standardized into adjusted score_i_. After standardization, adjusted scores showed reduced interference due to varied attendance. On the other hand, we found increased fluctuations in the adjusted score during the second half of the treatment program.

**Figure 5:**
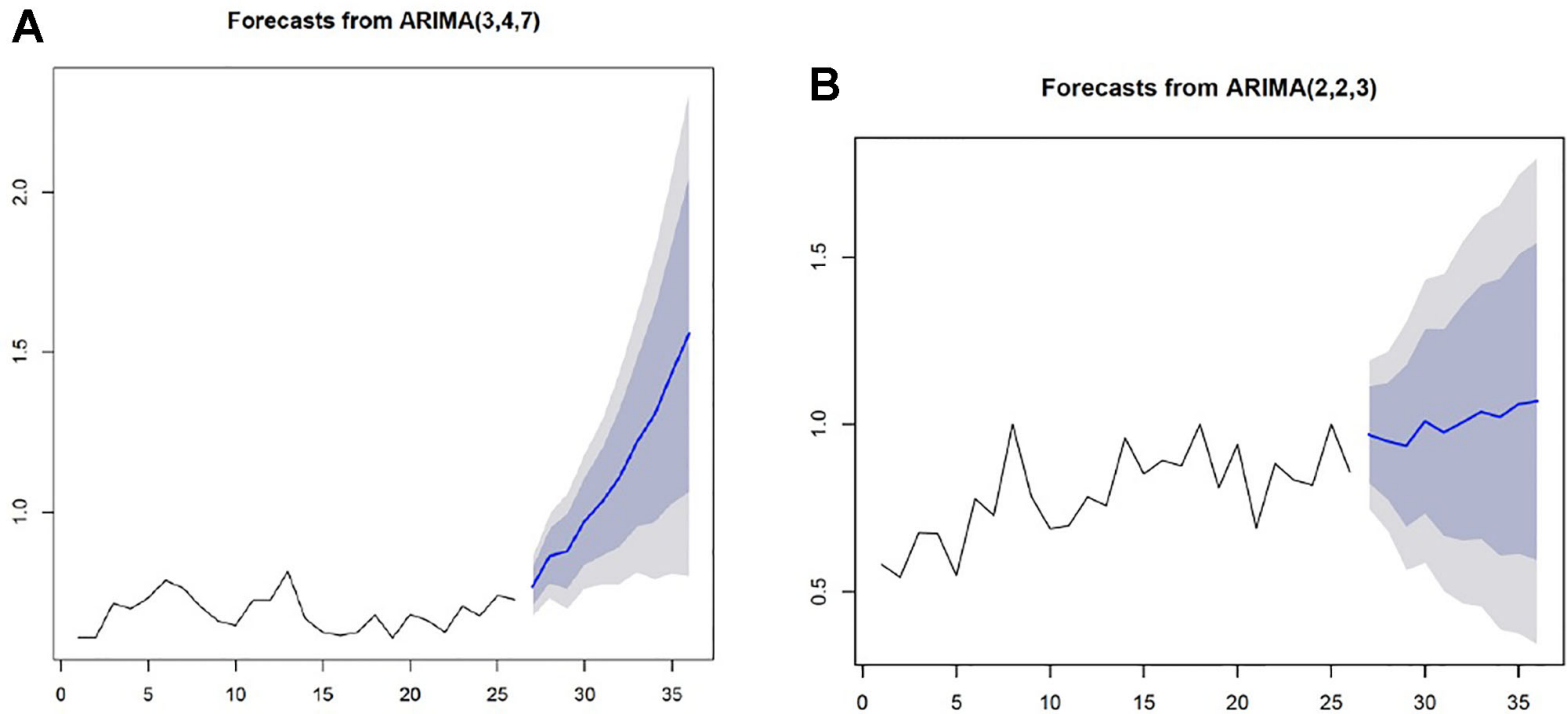
The ARIMA models predicting treatment outcomes for each phase **X**-**axis**: week, **Y**-**axis**: adjusted score **(A)** A sharply raised adjusted score indicating an increased number of negative urine tests was predicted for the high-demand treatment phase. **(B)** A slow and steady raised adjusted score was predicted for the low- demand treatment phase.

The first half of treatment revealed a trend spanning from week one to week 26, while the a second trend component was observed between week 27 and week 52. The coefficients of the lag variables for mean (p), lag variables for residual error term (q), and number of orders for ensuring stationary (d) were estimated at p = 3, d = 4, and q = 7 for the first half of the program, but were estimated at p = 2, d = 2, and q = 3 for the second half. Both models were stationary and tested as sufficiently fit for prediction (p < 0.05). Using the above two ARIMA models, we predicted an adjusted score that would reflect treatment outcomes during each program phase. Here, a sharply raised adjusted score was predicted during the high-demand phase, thus indicating an increasing number of negative urine tests. However, a slow and steady raised adjusted score was predicted for the low-demand phase (**Figure 5**).

## Discussion

We introduced a novel autonomic progress-bar tool to summarize urinalysis results for patients who were attempting to complete a program to treat stimulant and/or cannabis use disorder. We also developed an autonomic score calculator that totaled shared patient achievements. Resulting scores were analyzed as time-series data, thus revealing a trend that reflected compatible treatment demands.

This visual progress bar is currently a preliminary tool. However, elements of the intuitive design should be retained. For example, steadily increasing accumulated scores reflected persistent abstinence throughout the treatment course. Additional animated indicators may generate a curiosity-driven and pleasant experience. This is important because such factors are associated with the novelty-seeking trait (13, 14). We therefore believe that our progress bar will promote treatment engagement among patients with stimulant and/or cannabis use disorder.

Drug users may experience distress over the perception of lagging in progress. It is thus crucial to provide a virtual method for these individuals to generate forward-looking attitudes while preventing relapse (9, 15). Our visual progress bar offers a rewarding effect rarely obtained in contexts outside treatment programs. It may also offer experiences of increased mental engagement related to information-valuing functions, particularly those in the dopaminergic valuation systems (16, 18). From the perspective of neural encoding, information prediction during non-instrumental information seeking is not prone to error (16). This produces greater feelings of self-competence and promotes treatment adherence (19–20).

This study examined the effectiveness of a scoring approach through a visual progress bar in forecasting continually negative urine tests. We also examined performance at different treatment stages associated with changing demands and considered how the scoring system could be improved. As such, we plotted two ARIMA models to conduct time-series analyses for both the high and low treatment demand phases. We found obvious changes in scores based on treatment demands. For example, rapidly increasing scores reflected an increasing number of negative urine tests, which was forecast for the high-demand phase. On the other hand, a series of small, incremental score increases were predicted for the low-demand phase. Previous research has also shown that high-demand treatment is significantly correlated with continually negative urine testing (21). Here, the changes in scoring trends between the different treatment levels observed in this study supported existing evidence.

We found increased fluctuations in the adjusted score during the low-demand treatment phase. This is likely because the number of patients completing urine tests under the stringent biweekly schedule dwindled over time. For example, a given patient would complete urine testing during even-numbered weeks if following the attendance schedule exactly. However, missed appointments would disrupt the schedule, thus increasing the number of tests taken during odd weeks. As such, the numbers of patients attending during even and odd weeks were distributed more evenly over time. This was reflected by the fluctuating scores seen during the second half of the treatment program.

This study should be interpreted within the context of its limitations. First, we did not examine the validity of the progress bar through a clinical trial. This study did not compare a non- progress-bar-using group and a progress-bar-using group. Instead, we incorporated a quasi-experiment. As a result, the validity and reliability of this novel tool could not be determined. Second, patients were required to attend biweekly group psychotherapy sessions that began during their six months of treatment. As such, an instrumental effect due to this therapeutic influence may have been a confounding factor. Third, all patients in this study were subjected to mandatory treatment. This was because treatment approach for substance use offenders transited from detention-base to deferred prosecution, for recent two decades globally. Previous study proved the judicial-plus-therapeutic effect rather than judicial-alone effect (21).

In conclusion, we developed a novel progress-bar tool for use in motivating the completion of a stimulant and/or cannabis use treatment program. We also developed an autonomic score calculator that totaled shared patient achievements. Finally, a time-series analysis was conducted to examine the effectiveness of both the scoring approach and progress bar in forecasting continually negative urine tests. The open-source code for this free application is offered in the Supplementary Material section below and can easily be implemented for use in other substance-treatment services.

## Data Availability Statement

The raw data supporting the conclusion of this article will be made available by the authors, without undue reservation, to any qualified researchers.

## Ethics Statement

The studies involving human participants were reviewed and approved by Ethics Review Committee of Taichung Veterans General Hospital. Written informed consent for participation was not required for this study in accordance with the national legislation and the institutional requirements.

## Author Contributions

I-CC contributed to statistical analysis and drafting of the manuscript. GT contributed to the conception and interpretation of data. C-JC, T-HL and H-JL contributed to revising critically of the intellectual content.

## Funding

This work was supported by the Taichung General Veterans Hospital, Taiwan (Grant number: TCVGH-NHRI10602).

## Conflict of Interest

The authors declare that the research was conducted in the absence of any commercial or financial relationships that could be construed as a potential conflict of interest.
