# Supplementary material for: The Autonomic Progress Bar Motivates Treatment Completion for Patients of Stimulant Use Disorder and Cannabis Use Disorder
Source: Front Psychiatry. 2020 Jan 13;10:944. doi: 10.3389/fpsyt.2019.00944 (PMC6970339; doi:10.3389/fpsyt.2019.00944)
Supplement: Supplementary file 1 [file DataSheet_1.docx]

###Filename:ProgressBar.R

###Purpose: Plot trends of urine test results

#please set the directory according to your PC

#The input data follows the format:

# column 1: yyyy/mm/dd, such as 2017/4/12

# Column 2: 1 or -1 , depending on whether a negative or positive urinalysis result.

#import data

y=read.table(file="/Users/I-Chun/R/Day1.csv",header=TRUE,sep=",",col.names=c("DATE","RESULT"),stringsAsFactors=F)

x=y[,1]

x2=y[,2]

First_D=y[1,1]

First_date=as.Date(First_D)

d=numeric(length(x))

z=numeric(length(x))

w=numeric(length(x))

urine=numeric(length(x))

for (i in 1:length(x))

{

d[i]=y[i,1]

z[i]=as.Date(d[i])-First_date

w[i]=z[[i]]%/%7

}

wSmall=cbind(w,x2)

Total_visits=length(w)

TW=0:104

select=TW %in% w

Ze=rep(0,times=105)

wLarge=cbind(TW,Ze,select)

write(select,file="/Users/I-Chun/perl_tests/select.csv",ncolumns=1)

write.table(wLarge,file="/Users/I-Chun/perl_tests/wLarge.csv",row.names=F,col.names=F,sep=",")

write.table(wSmall,file="/Users/I-Chun/perl_tests/wSmall.csv",row.names=F,col.names=F,sep=",")

come=wSmall[,1]

count=numeric(length(TW))

score=numeric(length(TW))

for (j in 0:104)

{

if (j %in% come)

{

for (k in 1:length(come))

{

if (wSmall[k,1]==j) {score[j+1]=wSmall[k,2]}

}

}

else {score[j+1]=0}

}

score=score[-c(1)]

sum_score = numeric(length(score))

for (l in 1:length(score)) {

for (m in 1:l) {

sum_score[l] = sum_score[l] + score[m]

}

}

write.table(sum_score,file="/Users/I-Chun/R/d0.csv",row.names=F,col.names=F,sep=",")

#start plotting from which week

library(ggplot2)

Fig1=read.table(file="/Users/I-Chun/R/d0.csv",header=TRUE,sep=",",col.names=c("Week","Sum of Score"),stringsAsFactors=F)

X1=Fig1[,1]

Y1=Fig1[,2]

ggplot(data=Fig1)+geom_line(aes(x=X1,y=Y1))+geom_smooth(aes(x=X1,y=Y1))+labs(title="Patient1",x="Week",y="Sum of Score")+theme(panel.background=element_rect(fill="lightyellow",colour="lightyellow",size=0.5,linetype="solid"),panel.grid.major=element_line(size=0.5,linetype="solid",colour="white"),panel.grid.minor=element_line(size=0.25,linetype="solid",colour="white"))
